# Sound frequency dependence of duration mismatch negativity recorded from awake rats

**DOI:** 10.1002/npr2.12090

**Published:** 2019-12-01

**Authors:** Hiroyoshi Inaba, Hisaaki Namba, Hidekazu Sotoyama, Itaru Narihara, Eiichi Jodo, Hirooki Yabe, Satoshi Eifuku, Hiroyuki Nawa

**Affiliations:** ^1^ Department of Molecular Neurobiology Brain Research Institute Niigata University Niigata Japan; ^2^ Department of Systems Neuroscience Fukushima Medical University School of Medicine Fukushima Japan; ^3^ Department of Neuropsychiatry Fukushima Medical University School of Medicine Fukushima Japan

**Keywords:** auditory cortex, duration deviance, mismatch negativity, rats, sound frequency

## Abstract

**Aims:**

The brain function that detects deviations in the acoustic environment can be evaluated with mismatch negativity (MMN). MMN to sound duration deviance has recently drawn attention as a biomarker for schizophrenia. Nonhuman animals, including rats, also exhibit MMN‐like potentials. Therefore, MMN research in nonhuman animals can help to clarify the neural mechanisms underlying MMN production. However, results from preclinical MMN studies on duration deviance have been conflicting. We investigated the effect of sound frequency on MMN‐like potentials to duration deviance in rats.

**Methods:**

Event‐related potentials were recorded from an electrode placed on the primary auditory cortex of free‐moving rats using an oddball paradigm consisting of 50‐ms duration tones (standards) and 150‐ms duration tones (deviants) at a 500‐ms stimulus onset asynchrony. The sound frequency was set to three conditions: 3, 12, and 50 kHz.

**Results:**

MMN‐like potentials that depended on the short‐term stimulus history of background regularity were only observed in the 12‐kHz tone frequency condition.

**Conclusions:**

MMN‐like potentials to duration deviance are subject to tone frequency of the oddball paradigm in rats, suggesting that rats have distinct sound duration recognition ability.

## Abstract 2

We investigated the effect of sound frequency on mismatch negativity (MMN)‐like potentials to duration deviance in rats. The sound frequency was set to three conditions: 3, 12, and 50 kHz. As a result, MMN‐like potentials that depended on the short‐term stimulus history of background regularity were only observed in the 12‐kHz tone frequency condition.

## Graphical abstract

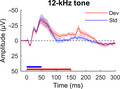

## 1 INTRODUCTION

The ability of the brain to detect deviations in the acoustic environment in humans can be assessed by auditory mismatch negativity (MMN).^1^, ^2^ MMN is triggered in the auditory cortex in response to rare deviations (eg, changes of pitch, duration, and intensity) from the regular pattern of standard sounds. It is calculated by subtracting the value of the event‐related potential (ERP) for standard sounds from that for deviant sounds. Reduction in MMN amplitudes is often seen in patients with schizophrenia.^3^, ^4^, ^5^, ^6^ MMN evoked by sound duration deviance has recently attracted attention as a biomarker for schizophrenia because reduced amplitudes have been identified from early schizophrenic stages.^7^, ^8^, ^9^, ^10^ Nonhuman animals, including rats, also show MMN‐like potentials that are characteristically similar to human MMN.^11^, ^12^ Therefore, MMN research in nonhuman animals can help reveal the neural mechanisms of MMN generation and its deficits.^13^ Currently, the few studies observing MMN evoked by duration deviance in rats have produced inconsistent results in the response latency window and polarity of MMN.^14^, ^15^, ^16^, ^17^ Acoustic stimuli used in these studies (1‐ to 4‐kHz tones) were located at the lower end of the hearing range in rats^18^; therefore, it was possible that the frequency of these sound stimuli was not suitable for MMN induction. Notably, a recent study using 7‐ to 18‐kHz sound stimuli observed large MMN‐like potentials to pitch changes.^19^ However, the effect of sound frequency on MMN‐like potentials to duration deviance remains unknown. Here, we recorded MMN‐like potentials on the primary auditory cortex of free‐moving rats to evaluate the effect of tone frequency on MMN evoked by duration deviance.

## 2 METHODS

The key resources table and the detailed method of data analysis are provided in Supporting Information.

### 2.1 Animals

Male Sprague‐Dawley rats (n = 11) were used and housed in the Niigata University Animal Facility under a reversed 12‐hour light/dark cycle (8:00 am OFF and 20:00 pm ON) at constant temperature and humidity. Solid food and water were available ad libitum.

### 2.2 Surgery and electrode placement

Surgery and electrode placement were performed as described previously.^20^ Rats (9‐10 weeks old) were anesthetized with pentobarbital (i.p., 65 mg/kg) or a combination anesthetic (i.p., 0.375 mg/kg medetomidine, 2 mg/kg midazolam, and 2.5 mg/kg butorphanol). Three miniature stainless‐steel bolts were used as electrodes for recording electrocorticography (ECoG). The bolts were screwed to the skull to contact the dura mater at the following points: the right primary auditory cortex (4.5 mm posterior to bregma; 8.0 mm lateral to the midline; and 4.2 mm below the top plane of the skull), the frontal sinus (as a reference), and the cerebellum (as a ground).^20^, ^21^ All lead wires from the electrodes were soldered onto a miniature connector. The connector was anchored to the cranium using dental cement. Rats received cefmetazole (i.p., 100 mg/kg) after surgery. Solid foods were replaced with softened food pellets (CMF sprout) to promote normal weight gain. Recording began at least 14 days after recovery from surgery.

### 2.3 Recording

Recording occurred during the dark cycle, which is the active phase in rats.^19^, ^20^ Each awake rat was placed in a transparent electrically shielded plastic box (dimensions 18 × 36 × 30 cm) during acclimation and recording sessions. ECoG signals from the electrodes were fed by wire into an amplifier (DAM‐80) and band‐pass filtered at 0.1‐1000 Hz. The amplified signals were digitized and recorded at a 4‐kHz sampling rate (Digidata1200B and Axoscope software).

### 2.4 Auditory stimulation

Auditory stimuli were generated by a dual‐channel signal generator (AFG‐3022) and delivered thorough a loudspeaker mounted above the recording chamber. The stimuli were 3‐, 12‐, and 50‐kHz sine tones (3‐ms rise and fall time) with the intensity set to 85 dB at 5 cm above the center of the chamber floor (SpectraPlus software).

**Figure 1:**
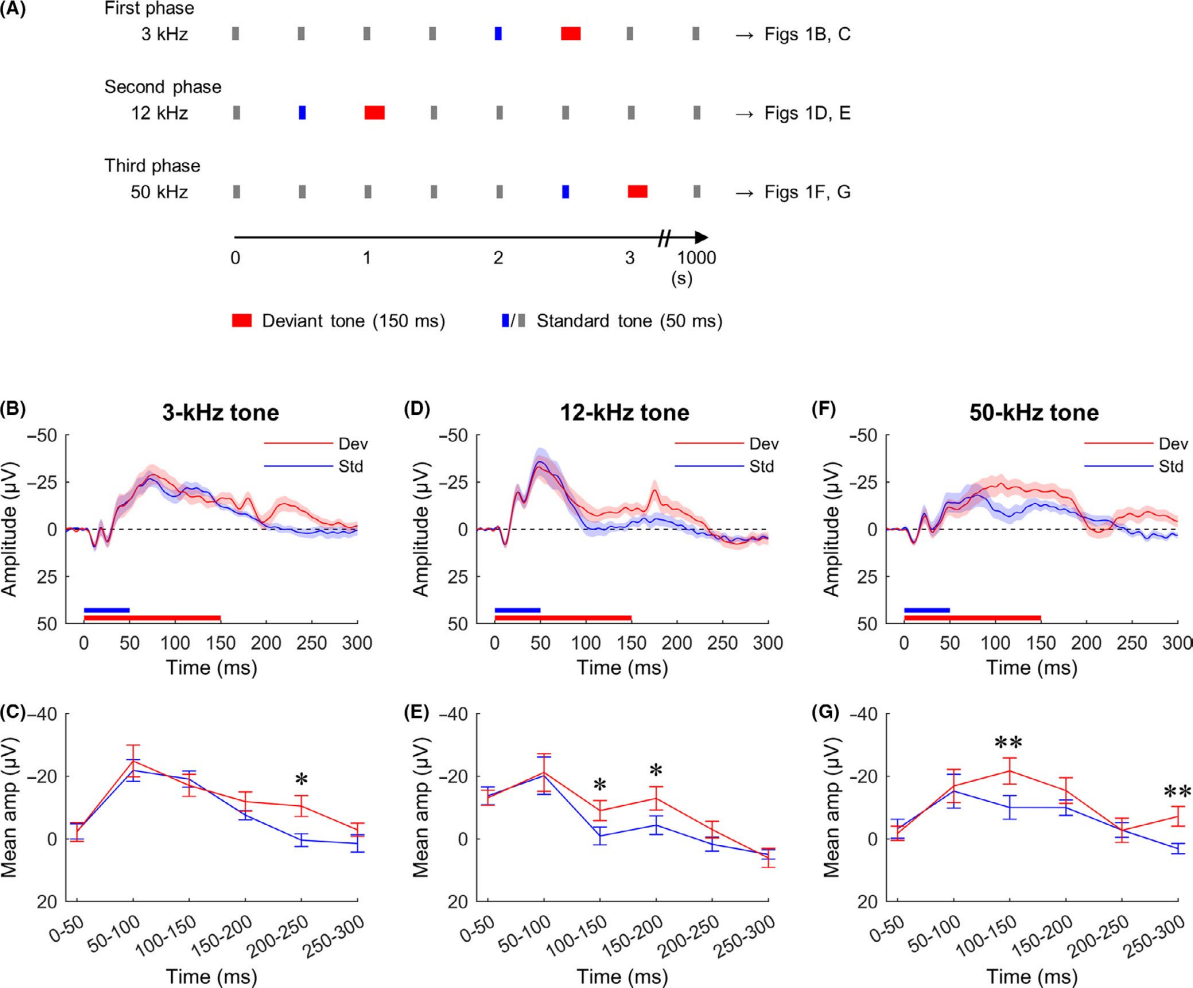
ERPs recorded from an electrode on the auditory cortex of rats. A, Stimulation sequences used in the experiment. The 3‐, 12‐, and 50‐kHz sine tones were used in the first, second, and third phases, respectively. Epochs were extracted for each deviant tone (red) and the standard tone immediately preceding it (blue). B, D, F, Grand averaged waveforms from the 3‐, 12‐, and 50‐kHz deviant and standard tones. C, E, G, The six consecutive time segments for mean response amplitudes from B, D, and F, Shaded areas around ERP traces and error bars denote SEM, n = 11 (*P < .05, **P < .01, repeated measures ANOVA followed by post hoc paired comparisons)

Tones were presented using an oddball paradigm with three experimental phases (3, 12, and 50 kHz, respectively), each with 2000 stimuli. In each phase, standard tones (50‐ms duration, 90% probability) and deviant tones (150‐ms duration, 10% probability) were presented by a microcontroller in pseudorandom order with a stimulus onset asynchrony of 500 ms (Figure 1A).

### 2.5 Data analysis

Data analyses were performed offline using MATLAB software. Acquired data were imported into the EEGLAB toolbox^22^ via Spike2 software. Epochs were extracted for each deviant tone and the immediately preceding standard tone (Figure 1A). Baselines were corrected by subtracting the mean value of the 0‐20 ms before tone onset.^15^, ^23^ Averaged time‐locked evoked potentials (ie, ERPs) were calculated separately for standard and deviant tones within each animal.

Repeated measures analyses of variance (ANOVA) with stimulus type (deviant and standard) and time segment (50‐100, 100‐150, 150‐200, 200‐250, and 250‐300 ms) as variables were run independently for each sound frequency.^14^ Tukey's post hoc tests were used to analyze the differences in time‐averaged amplitude between deviant and standard ERPs. In all statistical tests, *P* < .05 was considered as statistically significant.

## 3 RESULTS

We examined whether MMN responses to duration deviance differed depending on the tone frequency (3, 12, or 50 kHz). Figure 1B shows the grand average ERP waveforms to the 3‐kHz deviant and standard tones. The mean amplitudes of six consecutive 50‐ms time segments were calculated from the averaged ERP waveforms (Figure 1C). A repeated measure ANOVA revealed a significant interaction of stimulus type × time segment (stimulus‐type effect: *F*_1,10_ = 1.92, *P* = .20; time‐segment effect: *F*_5,50_ = 19.51, *P* < .001; and interaction: *F*_5,50_ = 5.59, *P* < .001). Post hoc paired comparisons revealed that the ERP to the deviant stimulus was significantly more negative than that of the standard stimulus at 200‐250 ms following the stimulus onset (*P* < .05).

The grand average ERP waveforms to the 12‐kHz deviant and standard tones are presented in Figure 1D. A repeated measures ANOVA revealed a significant interaction of stimulus type × time segment (stimulus‐type effect: *F*_1,10_ = 4.71, *P* = .06; time‐segment effect: *F*_5,50_ = 15.19, *P* < .001; and interaction: *F*_5,50_ = 4.58, *P* < .01; Figure 1E). Post hoc tests showed that the ERP to the deviant stimulus was significantly more negative than that of the standard stimulus at 100‐150 and 150‐200 ms (*P*’s < .05) following the stimulus onset.

Figure 1F shows the grand average ERP waveforms to 50‐kHz deviant and standard tones. The ANOVA revealed a significant interaction of stimulus type × time segment (stimulus‐type effect: *F*_1,10_ = 6.60, *P* < .05; time‐segment effect: *F*_5,50_ = 9.37, *P* < .001; and interaction: *F*_5,50_ = 6.24, *P* < .001; Figure 1G). Post hoc analyses revealed that the ERP to the deviant stimulus was significantly more negative than that of the standard stimulus at 100‐150 and 250‐300 ms (*P*’s < .01) following the stimulus onset.

**Figure 2:**
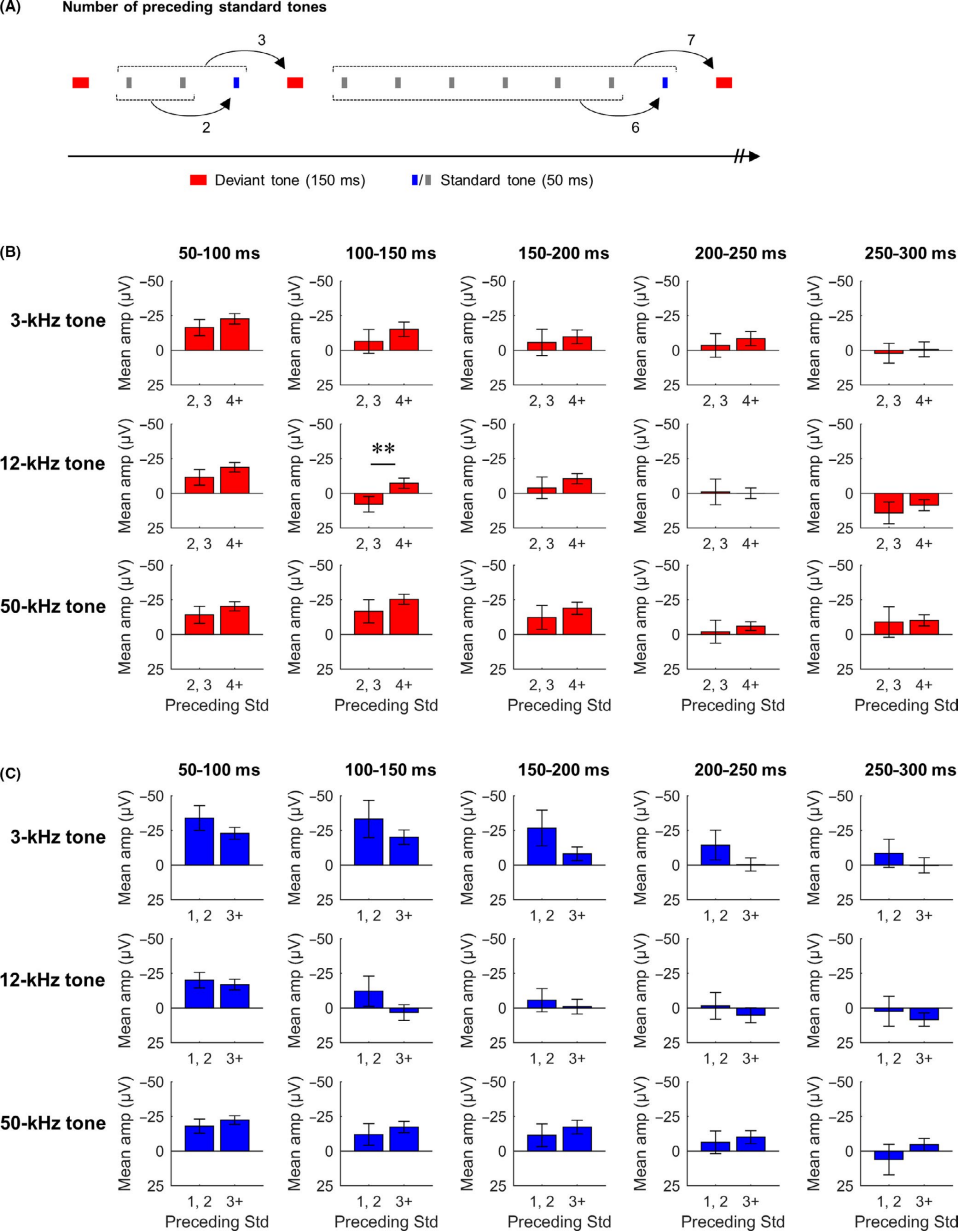
Sensitivity of ERPs to short‐term stimulus history. A, Examples of the number of preceding standard tones. The extracted epochs (red and blue) were separated by the number of preceding standard tones. B, Mean ERPs per time segment for deviant tones preceded by at most three (preceding std 2, 3) and at least four (preceding 4+) standard tones. C, Mean ERPs per time segment for standard tones preceded by at most two (preceding std 1, 2) and at least three (preceding 3+) standard tones. Error bars denote SEM, n = 11 (**P < .01, paired *t* test)

MMN amplitudes are thought to depend on short‐term stimulus history of background regularity.^24^, ^25^ It has previously been reported in rats that ERP amplitudes to pitch deviation show a marked increase when preceded by four or more standard tones.^16^ This phenomenon was examined in each time segment at all frequency conditions. In this analysis, the ERP amplitudes for deviants with four or more preceding standards were compared to those with less than four preceding standards (Figure 2A). Two‐tailed paired *t* tests revealed that the ERP for deviant tones in the 12‐kHz condition at 100‐150 ms increased in negative amplitude depending on the number of preceding standards (*df* = 10, *t* = 4.28, *P* < .01; Figure 2B, middle row). In contrast, no differences were seen in the 3‐ or 50‐kHz conditions (Figure 2B, top and bottom rows). Further, the ERP for standard tones that immediately preceded the deviant tones showed no changes depending on the number of preceding standards at all frequencies (Figure 2C).

These results indicate that MMN‐like potentials evoked by duration deviance only depend on the preceding stimulation history in the 12‐kHz tone frequency condition, suggesting that MMN‐like responses to duration deviance are subject to tone frequency in rats.

## 4 DISCUSSION

The purpose of our study was to evaluate the effect of sound frequency on MMN evoked by duration deviance in rats using three frequency tones. The results showed that MMN‐like potentials that were sensitive to short‐term stimulation history were observed at the 12‐kHz, but not 3‐ or 50‐kHz, tone frequencies.

A previous study has reported that MMN‐like potentials evoked by duration deviance are observed during the 100‐ to 160‐ms period following 3‐kHz tone onset using a similar paradigm as in our study (although, this study saw no dependence on short‐term stimulation history of background regularity).^16^ In the present study, no significant negative potentials were observed during that time period in the 3‐kHz condition. However, the tone intensity of our method (85 dB) was lower than that of the previous study (96‐105 dB). Therefore, the discrepancy may be due to such methodological differences.

In pitch MMN, increases in frequency elicit larger MMN amplitudes than decreases in frequency.^26^ It has been suggested that this phenomenon reflects the empirical salience of sounds.^27^ The 12‐kHz tone is relatively close to the tone frequency of rat 22‐kHz vocalizations that call attention to aversive stimuli.^28^ Thus, this tone may have high empirical salience. In addition, the hearing threshold for 12‐kHz tone is lower than that for 3‐ and 50‐kHz tones in various rats.^18^ These findings suggest that sound frequencies located near the center of the auditory range are suitable for the assessment of MMN‐like potentials to duration deviance in rats.

A limitation for this study is that there were no control conditions, such as the deviant‐standard‐reverse condition.^16^ 150‐ms duration tones were always used as deviant stimuli, and therefore, it cannot be denied that the MMN‐like potentials observed 100‐150 ms after stimulus onset reflected sensory‐driven activity rather than deviance‐related activity (“true” MMN). In addition, the order of the stimulation frequencies may also have affected the results, as the brain state probably changed during the measurement period. However, delta‐band power ratio (ie, arousal level) was not different throughout the period (Figure S1). Further studies would be needed to address these points.

In recent decades, MMN amplitude reduction has been considered to represent NMDA‐type glutamate receptor (NMDAR) hypofunction.^29^ Impaired glutamatergic neurotransmission is thought to be a significant cause of schizophrenia because noncompetitive antagonists of the NMDAR induce schizophrenia‐like symptoms including cognitive impairment.^29^ Our findings in nonhuman animals contribute to the establishment of MMN as a translational biomarker that reflects trait and state in psychiatric disorders, leading to the development of new drugs (eg, NMDAR‐targeted) and therapies.^30^

## CONFLICT OF INTEREST

The authors declare no conflict of interest.

## AUTHOR CONTRIBUTIONS

HI wrote the manuscript. HI and HNawa designed/performed the experiments and coordinated the work presented. HNamba, HS, IN, EJ, HY, and SE provided materials and commented on the manuscript.

## DATA REPOSITORY

All relevant data are included in Data S1.

## APPROVAL OF THE RESEARCH PROTOCOL BY AN INSTITUTIONAL REVIEWER BOARD

Not applicable.

## INFORMED CONSENT

Not applicable.

## REGISTRY AND THE REGISTRATION NO. OF THE STUDY/TRIAL

Not applicable.

## ANIMAL STUDIES

Ethical considerations for animal experiments are described in Supporting Information.
