# Supplementary material for: Sound frequency dependence of duration mismatch negativity recorded from awake rats
Source: Neuropsychopharmacol Rep. 2019 Dec 1;40(1):96–101. doi: 10.1002/npr2.12090 (PMC7292213; doi:10.1002/npr2.12090)
Supplement: Supplementary file 2 [file NPR2-40-96-s002.pdf]

Supporting Information

Figure S1

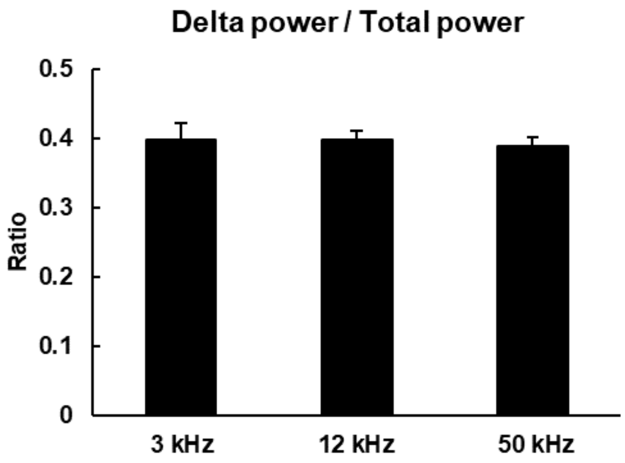

Figure S1: The ratio of the delta power (2 to 4 Hz) to the total power (2 to 100 Hz) of the ECoG during the pre-stimulus period (50 ms pre- to 20 ms pre-stimulus onset). ECoG power spectra were calculated for each stimulus-type (deviant and standard) and averaged for each individual with MATLAB and EEGLAB. Error bars denote *SEM*, $n = 11$ ($F_{1.32,13.25} = 0.25$, $p = 0.69$; one-way repeated ANOVA adjusted by Greenhouse-Geisser's epsilon).

Key Resources Table

**Reagent or resource**	**Source (identifier)**
Sprague-Dawley rats	Japan SLC, Shizuoka, Japan (MGI:5651135)
Pentobarbital	Kyoritsu Seiyaku, Tokyo, Japan
Medetomidine	Kyoritsu Seiyaku, Tokyo, Japan
Midazolam	Sandoz, Tokyo, Japan
Butorphanol	Meiji Seika Pharma, Tokyo, Japan
CMF sprout	Oriental Yeast, Tokyo, Japan
DAM-80	World Precision Instruments, Sarasota, FL
Digidata1200B	Axon Instruments, Foster City, CA
Axoscope software	Axon Instruments, Foster City, CA
AFG-3022	Tektronix, Beaverton, OR
SpectraPlus software	Pioneer Hill Software, Poulsbo, WA
MATLAB software	MathWorks, Natick, MA (RRID:SCR_001622)
EEGLAB toolbox	SCCN, La Jolla, CA (RRID:SCR_007292)
Spike2 software	CED, Cambridge, UK (RRID:SCR_000903)

Detailed Method of Data Analysis

Data analyses were performed offline using MATLAB software. Acquired data were imported into the EEGLAB toolbox[22] via Spike2 software. Data were down-sampled to 1 kHz and offline-filtered at 0.5–100 Hz. Epochs were extracted from 100 ms pre- to 500 ms post-stimulus onset for each deviant tone and the immediately preceding standard tone (Figure 1A). Epochs containing amplitudes exceeding ± 500 μV before baseline correction were rejected as artifacts (less than 10%). Baselines were corrected by subtracting the mean value of the 0–20 ms before tone onset[15,23]. Averaged time-locked evoked potentials (i.e. ERPs) were calculated separately for standard and deviant tones within each animal. The inter-stimulus interval (ISI) between deviant tone and its immediately following standard tone (350-ms ISI) was shorter than the ISI between standard tone and standard tone (450-ms ISI). Therefore, only epochs to standards preceded by at least one standard (and those immediately following deviants) were extracted. The mean (± *SEM*) number of epochs used for ERP calculation as follows: 3-kHz deviant, 190.18 ± 1.15; 3-kHz standard, 190.27 ± 1.30; 12-kHz deviant, 189.36 ± 1.49; 12-kHz standard, 189.27 ± 1.69; 50-kHz deviant, 190.91 ± 0.86; 50-kHz standard, 190.73 ± 0.85.

Repeated measures analyses of variance (ANOVA) with stimulus-type (deviant and standard) and time-segment (50–100, 100–150, 150–200, 200–250, and 250–300 ms) as variables were run independently for each sound frequency[14]. Tukey's post hoc tests were used to analyze the differences in time-averaged amplitude between deviant and standard ERPs. In all statistical tests, $p < 0.05$ was considered as statistically significant.

Animal Studies

All animal experiments were approved by the Animal Care and Use Committee of Niigata University. All efforts were made to minimize animal suffering and to reduce the number of animals used. The treatment of the animals was in accordance with the Act on Welfare and Management of Animals and the MEXT's Fundamental Guidelines for Proper Conduct of Animal Experiment and Related Activities in Academic Research Institutions.
